# Autophagy in Human Embryonic Stem Cells

**DOI:** 10.1371/journal.pone.0027485

**Published:** 2011-11-14

**Authors:** Thien Tra, Lan Gong, Lin-Pin Kao, Xue-Lei Li, Catarina Grandela, Rodney J. Devenish, Ernst Wolvetang, Mark Prescott

**Affiliations:** 1 Department of Biochemistry and Molecular Biology, Monash University, Clayton, Australia; 2 ARC Centre of Excellence in Structural and Functional Genomics, Monash University, Clayton, Australia; 3 Australian Institute for Bioengineering and Nanotechnology, University of Queensland, St. Lucia, Australia; 4 Center for Experimental and Molecular Medicine, Academisch Medisch Centrum, University of Amsterdam, Amsterdam, The Netherlands; University of Southern California, United States of America

## Abstract

Autophagy (macroautophagy) is a degradative process that involves the sequestration of cytosolic material including organelles into double membrane vesicles termed autophagosomes for delivery to the lysosome. Autophagy is essential for preimplantation development of mouse embryos and cavitation of embryoid bodies. The precise roles of autophagy during early human embryonic development, remain however largely uncharacterized. Since human embryonic stem cells constitute a unique model system to study early human embryogenesis we investigated the occurrence of autophagy in human embryonic stem cells. We have, using lentiviral transduction, established multiple human embryonic stem cell lines that stably express GFP-LC3, a fluorescent marker for the autophagosome. Each cell line displays both a normal karyotype and pluripotency as indicated by the presence of cell types representative of the three germlayers in derived teratomas. GFP expression and labelling of autophagosomes is retained after differentiation. Baseline levels of autophagy detected in cultured undifferentiated hESC were increased or decreased in the presence of rapamycin and wortmannin, respectively. Interestingly, autophagy was upregulated in hESCs induced to undergo differentiation by treatment with type I TGF-beta receptor inhibitor SB431542 or removal of MEF secreted maintenance factors. In conclusion we have established hESCs capable of reporting macroautophagy and identify a novel link between autophagy and early differentiation events in hESC.

## Introduction

Recent studies have not only demonstrated the evolutionary conservation of autophagy (Atg) gene function in vertebrates but also highlighted the involvement of the autophagy machinery in many aspects of tissue homeostasis. Despite its recognized roles in homeostasis, cancer, degenerative diseases and organelle turnover, and its essential role in preimplantation development of mouse embryos and cavitation of embryoid bodies, little is known about the role of autophagy in early human development Human embryonic stem cells (hESC) offer a unique window on the earliest differentiation events in early human development [1].

LC3 is currently the only molecular marker available for following the autophagosome in cells. GFP fused to LC3 is a well accepted approach to monitor autophagy whereby the appearance of green fluorescent puncta are indicative of the recruitment of LC3 to the forming autophagosomes [2], [3] We sought to establish and validate LC3-GFP reporter hESC lines. We used these cell lines to identify a novel link between autophagy and early differentiation of hESC.

## Results and Discussion

### Human Embryonic Stem Cell lines stably expressing GFP-LC3

**Figure 1:**
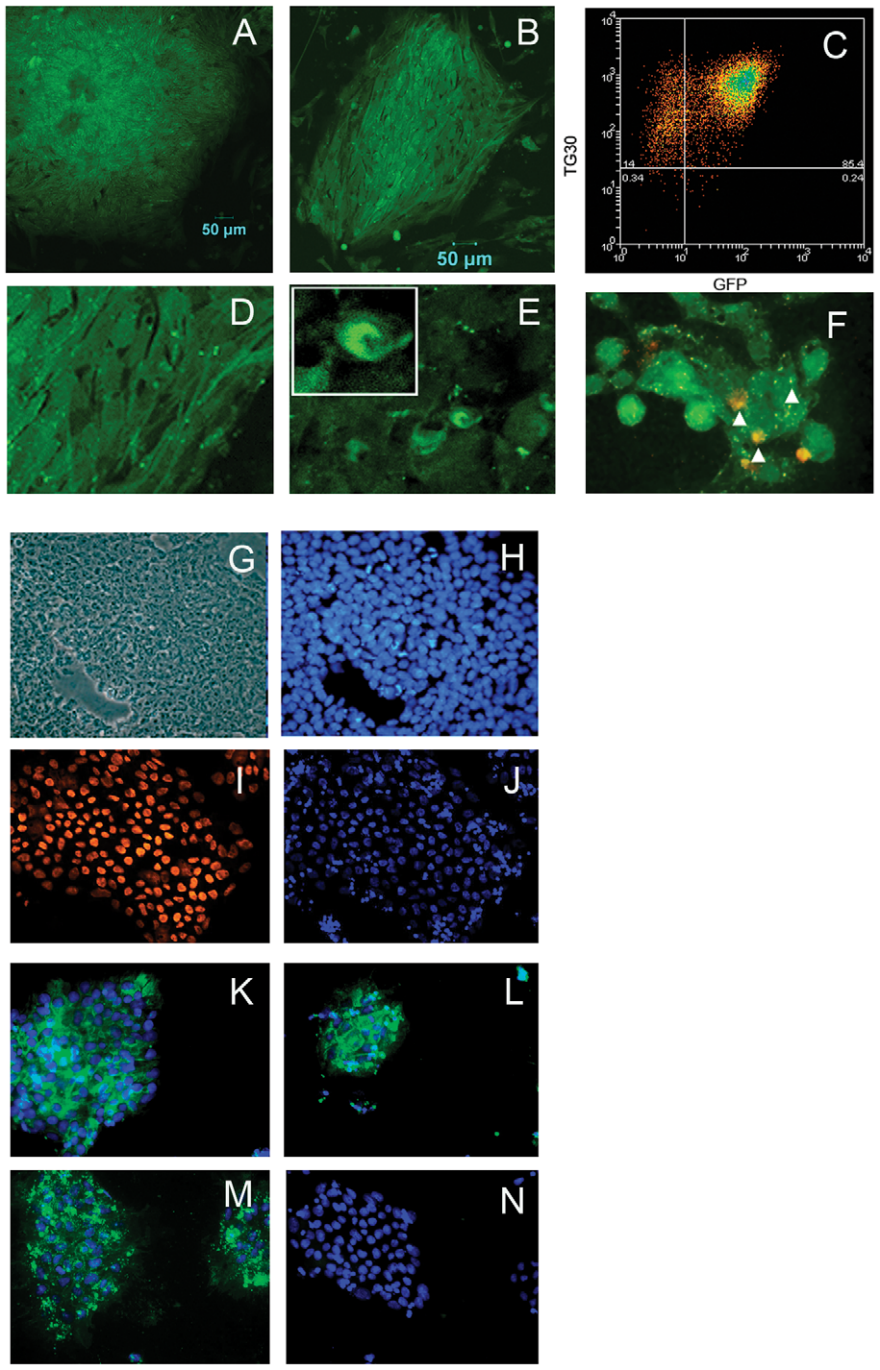
HES3 LC3-GFP cells are pluripotent. The majority of cells in an HES3-GFP-LC3 (A) or HES4-GFP-LC3 (B) colony express green fluorescence distributed evenly throughout the cytosol. Flow cytometric analysis of HES3-GFP-LC3 shows greater than 85% of the cells express GFP and the pluripotency marker TG30 (C). GFP-LC3 transduced hESC often show 1–2 bright fluorescent puncta (D) and the appearance of typical autophagosomes (E and insert). A subset of autophagosomes co-localise, as indicated by arrow heads, with the lysosomal membrane marker LAMP-1 (F). GFP-LC3 transduced hESC are small and densely packed when viewed under transmitted light (G) and show a high nuclear to cytoplasm ratio (H, cells stained for DNA with Hoechst). HES3-GFP-LC3 cells express the pluripotency markers Oct4 (I) and corresponding DAPI stained nuclei (J), TRA-181 (K), TRA-160 (L) and TG30 (M). No immunostaining was observed in isotype control stained cells (N).

Lentiviral transduction of an expression construct encoding GFP fused to LC3 and under the control of the constitutively active E1A (BOS) promoter was used to generate HES3 and HES4 hESCs (hereafter HES3-GFP-LC3 and HES4-GFP-LC3) that stably and constitutively express GFP-LC3 [4], [5]. After applying a Blasticidin selection regime the majority of cells in a population of HES3-GFP-LC3 (Fig. 1A) or HES4-GFP-LC3 (Fig. 1B) expressed GFP when viewed by fluorescence microscopy. Analysis by flow cytometry (Fig 1C) indicated that greater than 85% of HES3-GFP-LC3 cells express both GFP and the pluripotency marker TG30. At higher magnification (Fig. 1D) it was evident that GFP-LC3 transduced hESC, in addition to low GFP fluorescence in the cytosol, often display one or two bright fluorescent puncta that correspond in terms of size and appearance to autophagosomes (Fig. 1E) [3]. HES3-GFP-LC3 treated with rapamycin to induce autophagy and immunostained with an antibody against the lysosomal membrane protein LAMP-1 (Fig. 1F) showed green fluorescent puncta some of which co-localized with LAMP-1 labelling. This result indicates that the autophagosomes once formed are able to undergo fusion with lysosomes as would be required during the course of autophagy.

### Characterisation of HES3-GFP-LC3

HES3-GFP-LC3 displayed the typical small round and densely packed morphology of undifferentiated hESCs with a high nuclear-cytoplasm ratio and prominent nucleoli (Fig. 1G and 1H). These cells robustly expressed, Oct4 (Fig. 1I; DAPI staining for nuclei Fig 1J), TRA-181 (Fig. 1K), TRA-160 (Fig. 1L) and TG30 (Fig 1 M). Isotype control stained hESC displayed no immunoreactivity (Fig 1N). Importantly, cells displayed a normal karyotype (Fig. S1) indicating that lentiviral transduction and *in vitro* culture had not induced any detectable chromosomal rearrangements.

**Figure 2:**
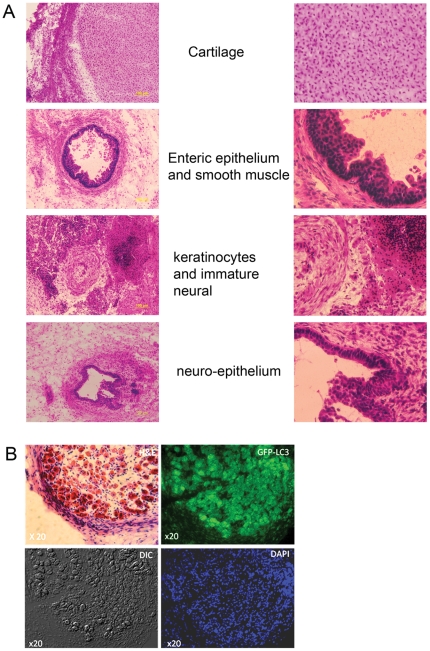
Teratoma sections derived from HES3 LC3-GFP cells and persistence of LC3-GFP transgene expression. (A), Representative sections of a 6 week old teratoma derived from HES3-LC3-GFP cells showed cell types representative of the three germlayers with each section shown magnified at right. (B), GFP fluorescence persists in teratomas of HES3-LC3-GFP cells. Representative images showing haemtoxylin/eosin staining and GFP fluorescence of two immediately adjacent serial sections of a HES3-LC3-GFP teratoma are shown. Brightfield and DAPI staining images corresponding to the GFP-fluorescence panel are shown. Arrow heads highlight autophagosomes in themagnified section (inset).

Six weeks after injection of HES3-GFP-LC3 into SCID mice teratomas were formed that showed evidence of differentiation into cell types representative of the three germlayers, including cartilage and muscle (mesoderm), glandular epithelium (endoderm) and keratinocytes, neuroepithelium and primitive neuronal cells (ectoderm) (Fig. 2A). GFP expression and fluorescent puncta were present in the cytosol of most of the differentiated derivatives of the teratomas (Fig. S2 A-D). The amount of GFP expressing tissue observed in these sections is consistent with the observation that 85% of undifferentiated HES3-GFP-LC3 express GFP. An example of hESC differentiated into ganglia is shown in Fig. 2B where at higher magnification autophagosomes could be clearly observed. Collectively these data suggest that hESCs over-expressing GFP-LC3 are pluripotent and that the persistence of transgene expression following long term differentiation *in vivo* will allow these cells to be used for investigating autophagy in most human cell types that can be differentiated from human embryonic stem cells. The LC3-GFP transgene is a commonly used reporter for the autophagosome and its expression in mice does not interfere with normal mouse development. We did not observe any evidence for the abnormal differentiation or proliferation of the hESC autophagy reporter lines generated in this study.

### Regulation of autophagy in HES3-GFP-LC3

**Figure 3:**
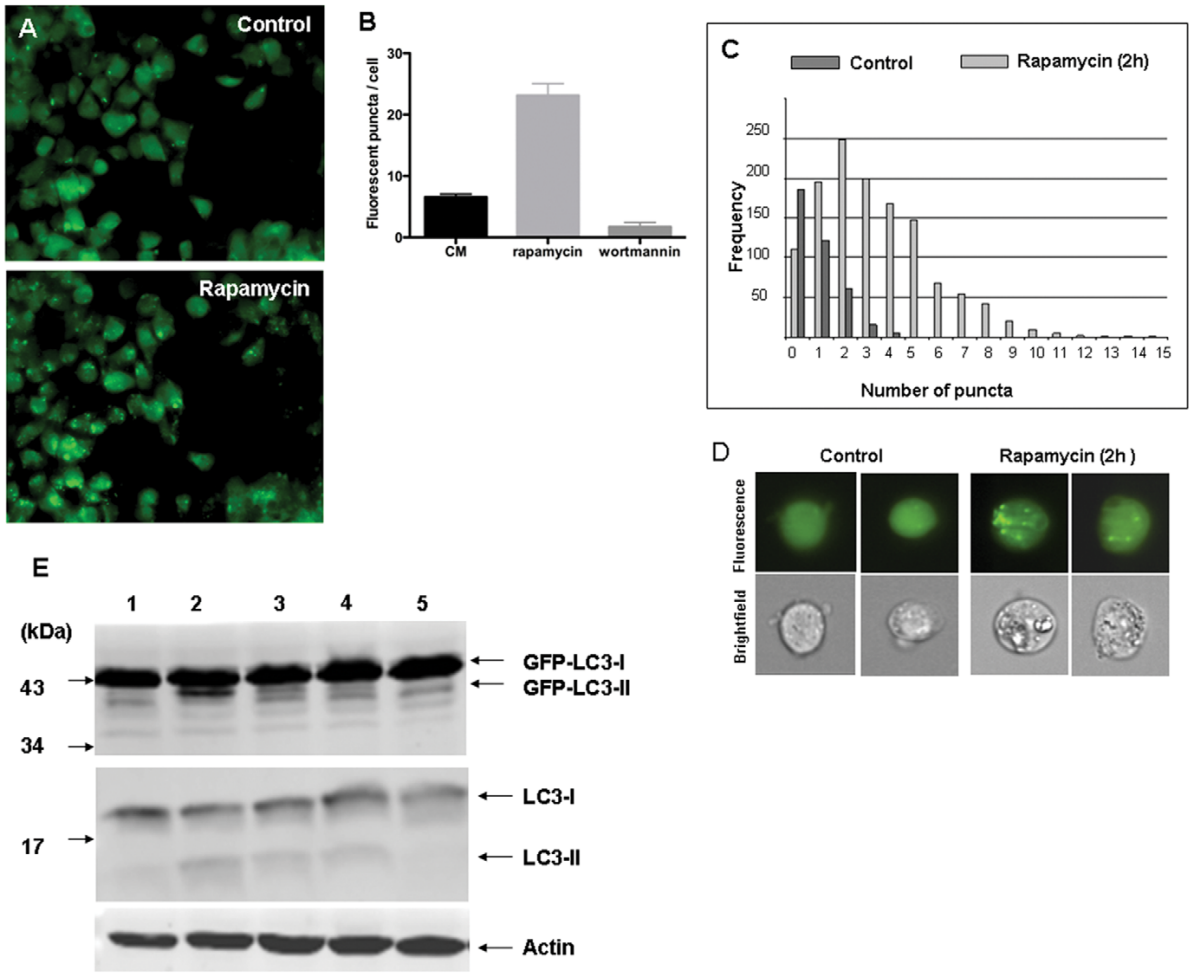
Regulation of autophagy in HES3 LC3-GFP cells. The number of fluorescent puncta observed by fluorescence microscopy in HES3 LC3-GFP cells under control conditions increased after incubation with rapamycin (200 nM) for 2 h (A). Numbers of fluorescent puncta in cells were determined in control cells and after treatment with rapamycin (200 nM), or wortmannin (150 nM) for 2 h (B). Using fluorescence microscopy over 100 cells were observed in 3 independent experiments. Cells with and without treatment with rapamycin (200 nM) for 2 h analysed by imaging flow cytometry (C). Numbers of fluorescent puncta in 10,000 individual cells from each sample were determined. Representative bright-field and fluorescence emission images for two individual cells from each sample are shown. The fluorescent puncta inside each cell are clearly visible (D). Cell lysates were prepared, subjected to SDS-PAGE and blots probed with a monoclonal antibody against LC3 (top and middle) or actin (lower panel) (E). Conditioned medium (CM) lane 1; CM + rapamycin for 2 h (200 nM), lane 2; unconditioned medium 3 days, lane 3; CM + SB431542 for 3 h (10 µM), lane 4; CM + wortmannin for 3 h (150 nM), lane 5.

Rapamycin, an inhibitor of mTOR, and wortmannin, a PI3-kinase inhibitor, are commonly used to induce or inhibit autophagy, respectively [6], [7]. The numbers of fluorescent puncta increased 4-fold from 6 to 23 per cell when feeder-free cultured HES3-GFP-LC3 cells were treated with rapamycin (200 nM) for 2 h (Fig. 3A & B). The average number of puncta was reduced to 2 per cell in cell treated with wortmannin (150 nM) (Fig. 3B). In agreement with these data the median number of puncta in HES3-GFP-LC3 cells treated with rapamycin was found to increase 4-fold from 1 (with a range from 1–7) to 4 (with a range from 1–27) puncta per cell when cell populations were analysed using imaging flow cytometry (Fig. 3A & D), a validated approach to quantify autophagy in large populations of cells [8].

The cytosolic precursor LC3 protein is rapidly converted to LC3-I by specific protease action [9], and upon induction of autophagy is recruited to the phagophore as the lipidated form, LC3-II. [3]. The LC3-I to LC3-II conversion can be used to monitor autophagy. SDS-PAGE immunoblot analysis of HES3-GFP-LC3 cell lysates showed the presence of LC3 immuno-reactive material with mobilities corresponding to GFP-LC3-I (M_r_ 45,000) and endogenous LC3-I (M_r_ 18,000) together with smaller amounts of GFP-LC3-II (M_r_ 40,000) and endogenous LC3-II (M_r_ 13,000) (Fig. 3E). Cells treated with rapamycin showed a significant increase in both the GFP-LC3-I/GFP-LC3-II and endogenous LC3-I/LC3-II ratios. Treatment of cells with wortmannin reduced these ratios. We conclude that autophagy occurs at a basal level in undifferentiated hESC and is subject to mTOR and PI3-kinase control, similar to other cell types [10], [11].

###  A novel link between autophagy and differentiation of hESC

**Figure 4:**
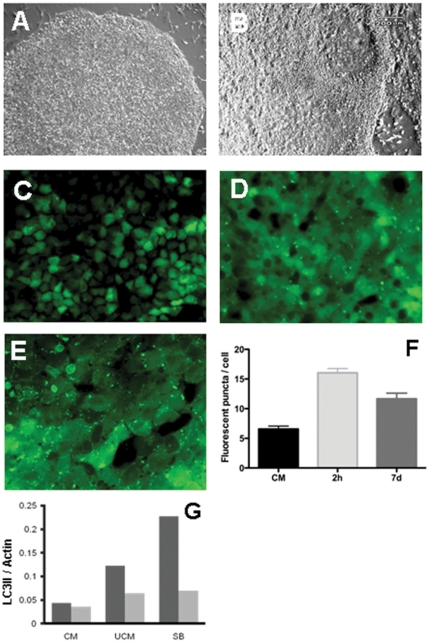
Spontaneous and induced differentiation promotes autophagy in HES3 LC3-GFP cells. Cells were maintained in either conditioned medium (CM, panels A, C) or unconditioned medium (B, D). Bright field images of an individual colony at low magnification (A, B) and fluorescence images are shown. Cells maintained in CM supplemented with SB431542 (10 µM) were imaged after 3 hours incubation (E). Fluorescent puncta were counted in cells for three independent experiments after h and 7 days of incubation in the presence of SB431542 (F). Analysis of western blot data (see Fig 3) was used to calculate the GFP-LC3II/Actin and LC3II/Actin ratios after 3 day culture in unconditioned medium (UCM) and SB431542 treatment for 3 hours (G).

Depriving hESC of MEF secreted factors is known to lead to differentiation [12], [13]. HES3-GFP-LC3 cells cultured for 3 days under feeder-free conditions in unconditioned medium display an increase in both the number of fluorescent puncta (Fig. 4 D) as compared to HES3-GFP-LC3 cells in conditioned medium (Fig. 3C), and LC3-II processing (Fig. 3E, lane 2 and Fig. 4G). This increase in autophagy is most likely related to the cellular remodelling events that accompany spontaneous differentiation of hESC. Remarkably, acute induction of hESC differentiation with 10 µM SB431542, a TGF β rec II inhibitor [14], [15], leads to a rapid increase in the number of fluorescent puncta (Fig. 4 E & F) as early as 2 hours after addition of the inhibitor and increased LC3-II (Fig. 3E, lane 4), after 3 hours. SB431542 treatment did not result in autophagy induction in differentiated hESC, murine fibroblasts or RAW264.7 macrophages indicating this is not due to TGF-β receptor inhibition *per se* (data not shown). Our data therefore suggest a link between autophagy and early small molecule-enforced differentiation of hESC. To our knowledge an increase in autophagy in human embryonic stem cells during these earliest steps of differentiation has thus far not been reported. mTOR signaling was previously shown to be important for proliferation of undifferentiated embryonic stem cells [16]–[18], and to enhance osteoblastic differentiation of hESC following a 5–7 day exposure to rapamycin. However, the focus in those studies was on the mTOR-S6 kinase axis rather than the autophagy inducing effect of rapamycin. Gene knock out studies in mice have demonstrated that autophagy plays a role in visceral endoderm and neural tube differentiation [19], [20] and is a major route for lysosomal protein degradation [21]. Murine ES cells (mESC) were found to display an increase in LC3I to LC3II conversion, indicative of autophagy, 3 to 6 days after differentiation as embryoid bodies. Indeed, mESC in which the gene for Beclin1, a regulator of autophagy, was inactivated display a failure in embryoid body cavitation and an accumulation of apoptotic cells in 3 to 6 day old Beclin1 null murine embryoid bodies [20]. These data showed that autophagy is involved in the cellular remodelling events following lineage specification and acquisition of morphological and functional differences of differentiated cell types. In contrast our data show an increase in LC3 processing as early as 3 hours after addition of SB431542 (Fig 3E) to human ESC, suggesting that autophagy induction and early differentiation events in hESC may be functionally linked. Despite our observation that no increase in autophagy in fibroblasts, macrophages or differentiated hESC is observed following SB431542 exposure and the published data indicate that TGFβ-receptor activation by TGFβ leads to increased autophagy [22], [23], we cannot formally exclude the possibility that TGF β rec II inhibition somehow leads to mTOR inhibition in a pluripotent hESC specific manner.

It is unlikely that the observed increase in autophagy during early differentiation of hESC is due to a starvation response as the culture medium on differentiating hESC cultures was changed daily. Although our data do not allow us to determine whether autophagy is upstream, concomitant with, or downstream of differentiation we speculate that autophagy may play an upstream regulatory role in hESC differentiation through degradation of pluripotency regulating protein complexes.

### Summary

In this study we have established hESCs expressing the GFP-LC3 reporter and for the first time demonstrate the occurrence of autophagy in hESCs. Using the hESC autophagy reporter lines generated in this study we provide preliminary evidence for a previously unrecognized link between autophagy and early differentiation of hESC.

## Materials and Methods

### Cell culture

Human ES cell lines HES-3 or HES-4 stock cultures were maintained and expanded as described [24]–[26] Feeder-free cultures were maintained as described [27].

### Lentiviral transduction

Lentivirus containing pLenti BOS-LC3-IRES-GFP vector was produced as described [28]. Feeder-free cultured HES-3 or HES-4 hESCs were transduced with BOS-LC3-IRES-GFP lentivirus (M.O.I. = 5) in the presence of polybrene (6 µg/ml). Selection with blasticidin (5 µg/ml) was maintained for 10 days and stably transduced blasticidin resistant hESC colonies expanded and propagated as described [28].

### Western Blotting

Polypeptides were separated on 12% SDS-polyacrylamide gels. Western blots were probed with mouse monoclonal antibodies against LC3 (NanoTools) (1∶500) or Actin (1∶1000) and visualized using AlexaFluor 488-conjugated goat-anti-mouse IgG (Molecular Probes) (1∶2000) [29].

### Flow cytometry

hESC's were harvested by collagenase digestion and single cell suspensions prepared using Cell Dissociation Solution (Sigma). Conventional flow analysis of CD9/TG30 expression was performed using a Cytomics FC 500 series flow cytometer (Beckman Coulter) [28].

For imaging flow analysis images of single cells were acquired using an ImageStream multispectral imaging flow cytometer (Amnis Corporation, Seattle, WA) with 488nm laser light excitation. Cell populations were gated for both GFP emission and single cells, and image data processed for counting of fluorescent puncta. Using IDEAS v3.0 software (Amnis Corporation, Seattle, WA) the threshold parameters were applied to the default mask of the representative cells according to the manufacturer's instructions. At least 10,000 cells were analysed in duplicate for each sample.

### Immunofluorescence staining

Immunostaining using antisera against TRA-1–60 (1∶100, Millipore MAB4360), TRA-181 (1∶100, Millipore), Oct3/4 (1∶50, Santa Cruz sc-5279) or LAMP-1 (1∶250, Development Studies Hybridoma Bank, University of Iowa, IA) was performed as described [29]. Images were acquired using wide-field fluorescence microscopy and processed using Volocity software (Olympus).

### Teratoma formation

Analyses of teratomas formed by GFP-LC3 transduced hESC lines were performed as described [29] with the exception that freshly harvested teratomas were embedded in OCT compound for cryosectioning. Adjacent serial sections were either: (i) fixed with formaldehyde and stained with Heamatoxilin/Eosin, (ii) imaged for GFP expression, or (iii) fixed with paraformaldehyde, stained with antibodies against GFP and imaged for the fluorescent conjugated secondary antibody.

## Supporting Information

Figure S1 **Karyotype analysis of HES3 LC3-GFP cells and HES4 LC3-GFP cells.** HES3 LC3-GFP cells and HES4 LC3-GFP hESC show normal karyotypes and G-banding.
(TIF)

Figure S2 **H&E staining and GFP fluorescence in adjacent sections of a teratoma derived from HES3 LC3-GFP cells.** Representative pairs of serial cryosections (A,B; C,D) of a 6 week old teratoma derived from HES3-LC3-GFP cells were either fixed and stained with haemtoxylin and eosin (A and C) or imaged directly for fluorescence due to GFP (B and D). Portions of A and B section are shown enlarged in C and D.
(TIF)
