# Supplementary figures and images for: Autophagy in Human Embryonic Stem Cells
Source: PLoS One. 2011 Nov 14;6(11):e27485. doi: 10.1371/journal.pone.0027485 (PMC3215747; doi:10.1371/journal.pone.0027485)

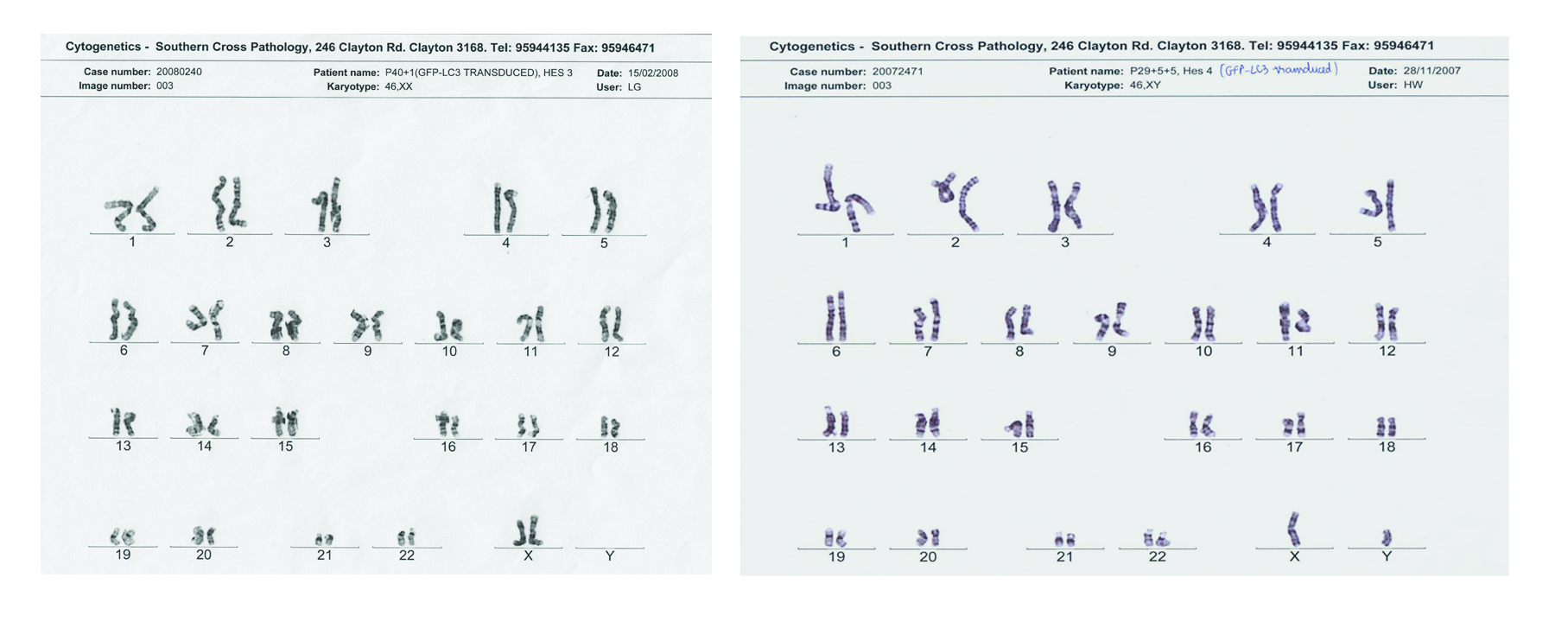

Supplement: Figure S1 — Karyotype analysis of HES3 LC3-GFP cells and HES4 LC3-GFP cells. HES3 LC3-GFP cells and HES4 LC3-GFP hESC show normal karyotypes and G-banding. (TIF) [file pone.0027485.s001.tif]

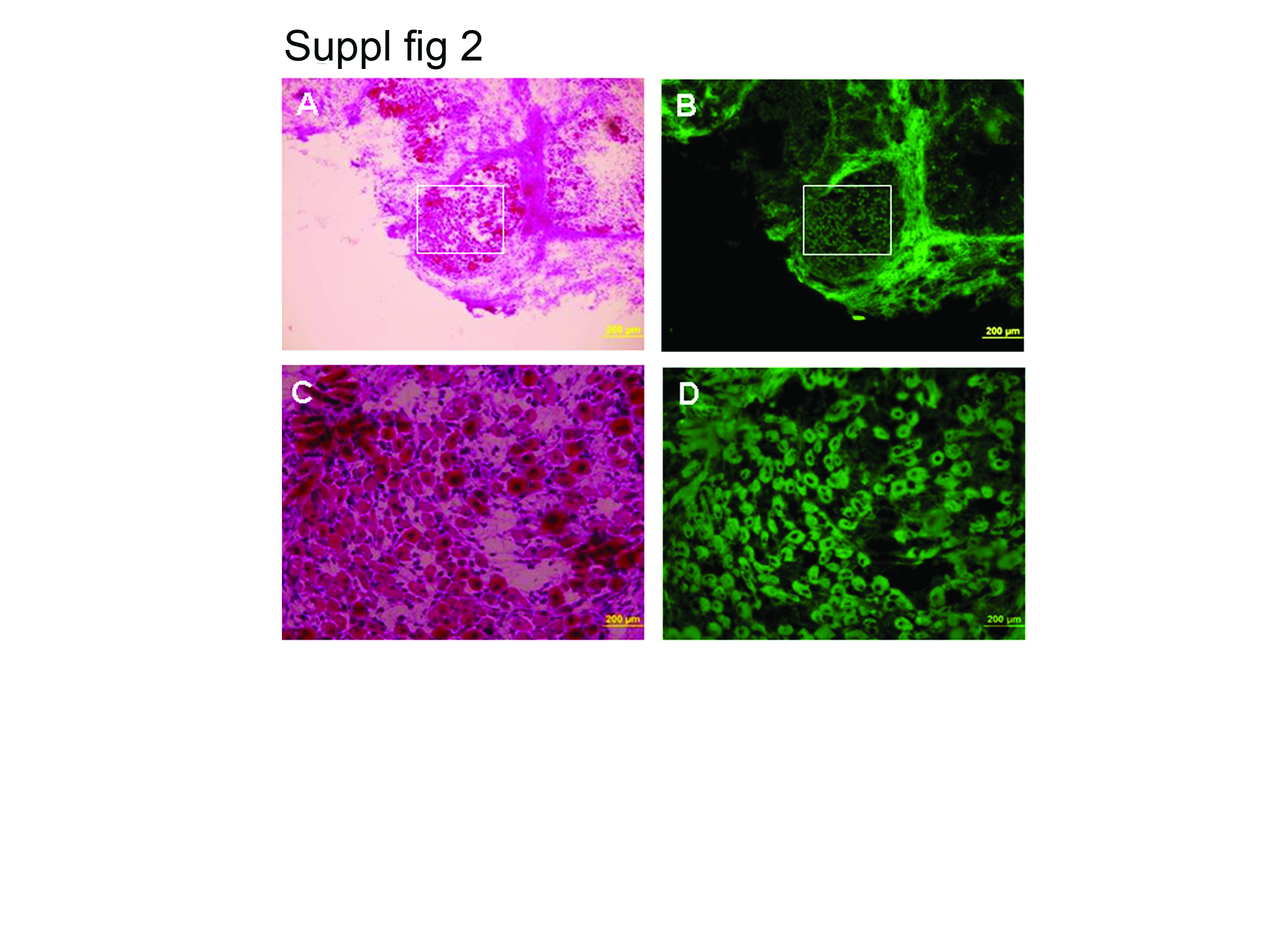

Supplement: Figure S2 — H&E staining and GFP fluorescence in adjacent sections of a teratoma derived from HES3 LC3-GFP cells. Representative pairs of serial cryosections (A,B; C,D) of a 6 week old teratoma derived from HES3-LC3-GFP cells were either fixed and stained with haemtoxylin and eosin (A and C) or imaged directly for fluorescence due to GFP (B and D). Portions of A and B section are shown enlarged in C and D. (TIF) [file pone.0027485.s002.tif]
